# Dynamic Hierarchical Energy-Efficient Method Based on Combinatorial Optimization for Wireless Sensor Networks

**DOI:** 10.3390/s17071665

**Published:** 2017-07-19

**Authors:** Yuchao Chang, Hongying Tang, Yongbo Cheng, Qin Zhao, Baoqing Li, Xiaobing Yuan

**Affiliations:** 1 Science and Technology on Microsystem Laboratory, Shanghai Institute of Microsystem and Information Technology, Chinese Academy of Sciences, Shanghai 201800, China; yuchaoc@mail.ustc.edu.cn (Y.C.); tanghy@mail.sim.ac.cn (H.T.); cyb@mail.sim.ac.cn (Y.C.); qinzhao21@mail.sim.ac.cn (Q.Z.); libq@mail.sim.ac.cn (B.L.); 2 University of Chinese Academy of Sciences, Beijing 100049, China

**Keywords:** wireless sensor networks (WSNs), hierarchical network structure, feasible routing sets, maximum minimum criterion, combinatorial optimization, balancing energy consumption

## Abstract

Routing protocols based on topology control are significantly important for improving network longevity in wireless sensor networks (WSNs). Traditionally, some WSN routing protocols distribute uneven network traffic load to sensor nodes, which is not optimal for improving network longevity. Differently to conventional WSN routing protocols, we propose a dynamic hierarchical protocol based on combinatorial optimization (DHCO) to balance energy consumption of sensor nodes and to improve WSN longevity. For each sensor node, the DHCO algorithm obtains the optimal route by establishing a feasible routing set instead of selecting the cluster head or the next hop node. The process of obtaining the optimal route can be formulated as a combinatorial optimization problem. Specifically, the DHCO algorithm is carried out by the following procedures. It employs a hierarchy-based connection mechanism to construct a hierarchical network structure in which each sensor node is assigned to a special hierarchical subset; it utilizes the combinatorial optimization theory to establish the feasible routing set for each sensor node, and takes advantage of the maximum–minimum criterion to obtain their optimal routes to the base station. Various results of simulation experiments show effectiveness and superiority of the DHCO algorithm in comparison with state-of-the-art WSN routing algorithms, including low-energy adaptive clustering hierarchy (LEACH), hybrid energy-efficient distributed clustering (HEED), genetic protocol-based self-organizing network clustering (GASONeC), and double cost function-based routing (DCFR) algorithms.

## 1. Introduction

With increasing advances in wireless communication systems, electronics and sensing technologies, wireless sensor networks (WSNs) have recently become an important, indispensable platform for a great number of vital surveillance and control applications [1]. A WSN consists of hundreds to thousands of tiny sensor nodes that are equipped with the same hardware structure and software capabilities (such as sensing, data processing, communication, etc.) to perform distributed sensing tasks and to transmit or forward information gathered to the base station (BS). Due to flexible use, a convenient layout and a low cost, WSNs have been widely used in military reconnaissance, environmental monitoring, smart home, industrial control, and other application fields [2,3,4,5].

With much more sensor nodes, WSNs are different from the traditional network in many aspects, including the architecture, addressing the method, the communication mode, the routing structure, etc. With the flat routing structure (i.e., a single-layer planer structure), the routing protocol is presently one of the key technologies in WSNs [5,6]. In general, as sensor nodes are powered by limited energy, energy conservation is commonly considered the most key challenge to guarantee the connectivity of WSNs and to extend the lifetime of sensor nodes, especially when the sensing field is inaccessible and the battery cannot be replaced [7]. Hence, to improve WSN longevity, routing protocols that aim to optimize power usage should become involved to balance the energy consumption of sensor nodes [4,8].

Topology control is one of the critical strategies for routing protocols in WSNs to improve network longevity. This is dedicated to periodical restoration of network topology in order to achieve the desired connectivity and ensure sufficient routes [9]. Network topology is the foundation of the better connectivity and the optimal route, while it also contributes to effective routing protocols for controlling the network route. Effective routing protocols help to improve WSN longevity by determining which sensor nodes should participate in the network routing operation and which should not at each transmission round [10].

Towards improving WSN longevity, we pay more attention to topology control to form the optimal network structure. However, we abandon the clustering mechanism and propose a dynamic hierarchical protocol based on combinatorial optimization (DHCO) to establish a feasible routing set for each sensor node. The feasible routing set is equivalent to a solution space consisting of many valid routes. The optimal route is obtained from the solution space by using the maximum–minimum criterion, which can be formulated as a combinatorial optimization problem. For the consideration of balancing the energy consumption of sensor nodes, the essence of the DHCO algorithm is to obtain the optimal route for each sensor node from its corresponding feasible routing set. Differently to state-of-the-art WSN routing algorithms (i.e., low-energy adaptive clustering hierarchy—LEACH, hybrid energy-efficient distributed clustering—HEED, genetic protocol-based self-organizing network clustering—GASONeC, double cost function-based routing—DCFR, etc.), in selecting the cluster head or the next hop node on the basis of the node energy property or the node distance property, the DHCO algorithm employs a hierarchy-based connection mechanism to construct a hierarchical network structure, and utilizes the combinatorial optimization theory to establish the feasible routing set for each sensor node, taking advantage of the maximum–minimum criterion to obtain their optimal route to the base station; this contributes to balancing the energy consumption of sensor nodes and improving WSN longevity. The remainder of this paper is organized as follows. We present related work on routing protocols to improve WSN longevity in Section 2. Section 3 focuses on the system model of WSNs, including the network model and the sensor energy model. Section 4 addresses the model of our proposed protocol. Experimental results in comparison to state-of-the-art WSN routing algorithms are discussed in Section 5. Finally, Section 6 gives conclusions.

## 2. Related Work

Clustering routing protocols have been widely researched in WSNs, and have achieved very fruitful results. In the clustering network structure, sensor nodes are divided into several clusters; each cluster is a node set of a cluster head (CH) and a plurality of member sensor nodes. The CH is not only accountable for managing member sensor nodes, but it also presides over data aggregation and data forwarding between clusters. The earliest classical clustering protocol of WSNs is the LEACH protocol [11], which optimizes the network energy expenditure by creating clusters dynamically. It was proposed initially as a distributed and single-hop clustering protocol, where sensor nodes made autonomous decisions without any centralized control. CHs are randomly selected by a predefined probability *p* (i.e., the desired percentage of CHs in WSNs), which forms clusters accordingly. However, the randomized process fails to select certain sensor nodes (such as those with more residual energy or in a better position) as CHs, which results in unevenly distributed CHs and premature energy depletion of these sensor nodes [12]. Consequently, the key step is the election of CHs, which requires taking into consideration plenty of factors, such as the node energy property, the node distance property, etc.

In the past decades, many classical WSN routing protocols utilizing the node energy property to search for the optimal clustering structure have arisen. A representative routing protocol is the stable election protocol (SEP) [13], which is a heterogeneous-aware protocol prolonging the time interval before the death of the first sensor node. The death of the first sensor node is crucial for many applications in which the feedback from WSNs must be reliable. Considering the node residual energy, the SEP algorithm selects CHs by using weighted election probabilities. Later, developed distributed energy-efficient clustering (DDEEC) [14] improved upon the SEP algorithm by selecting CHs with a probability based on the ratio between the residual energy of each sensor node and the average energy of the network. In the DDEEC algorithm, sensor nodes with a high residual energy have more chances to be CHs than low-residual energy nodes. Recently, differently to the previous WSN clustering protocols, Ren et al. [15] proposed an analytic model to estimate WSNs’ lifetimes, and developed the DCFR protocol that selects the next hop based on the node residual energy and the energy consumption rate. SEP, DDEEC, and DCFR algorithms pay attention to the node energy property and ignore other key factors of sensor nodes (i.e., the node distance property, etc), which is not optimal to maximize the WSNs longevity.

Alternatively, the node distance property is another significant aspect of developments upon the LEACH algorithm. At present, a great many efficient routing protocols based on the node distance property have arisen, such as the power-efficient gathering in sensor information systems (PEGASIS), energy-efficient unequal clustering (EEUC), and gateway based energy-efficient routing protocol (M-GEAR) algorithms. PEGASIS [16] is an optimal chain-based protocol that is an improvement upon the LEACH algorithm. In the PEGASIS algorithm, the sensor node chain for each sensor node is formed by communicating with a closer neighbor, and sensor nodes of a cluster rotate to transmit data to the BS. Similarly, in [17], Li et al. proposed an EEUC mechanism partitioning sensor nodes into clusters of unequal size. In the EEUC algorithm, clusters closer to the BS have smaller sizes than those farther away, which can preserve energy for WSNs. Like the EEUC algorithm, M-GEAR [18] is also a location-based protocol, and divides sensor nodes into four logical regions on the basis of their location in the sensing field. For the M-GEAR algorithm, the center of the sensing field has a gateway sensor node if the distance from a sensor node to the BS is greater than a predefined threshold and the communication between the sensor node and the BS is built on the basis of clusters; otherwise, the sensor node directly communicates with the BS. The PEGASIS, EEUC, and M-GEAR algorithms only consider the node distance property, which cannot completely represent characteristics of WSNs, and fails to effectively balance a greater energy consumption of the sensor nodes. Hence, the issue of maximizing WSN longevity is unsolved. More recently, joint optimization of the node energy and node distance properties has been extensively explored to identify the optimal network structure, and has triumphantly inspired people to put forward many effective routing protocols to improve WSN longevity. A HEED algorithm [10] is the typical representative routing protocol. Specifically, the HEED algorithm synthetically considers the mixture of the node residual energy and a secondary parameter (i.e., the sensor node proximity to its neighbors or the sensor node degree) to periodically select CHs. Despite that the HEED algorithm has improved the speed of clustering and reduced the cost of communication between clusters, it excludes some sensor nodes from joining into any clusters because of the competition between clusters [19]. Leader election with load balancing energy (LELE) [20] is a routing protocol promoted over the LEACH algorithm, which selects CHs by considering the amount of node residual energy and the distance between the sensor node and its neighbouring nodes. Although the LELE algorithm slightly improves the WSN longevity, the issue of unevenly distributed CHs still exists, which is not useful for maximizing WSN longevity. Furthermore, a framework to dynamically optimize clusters of WSNs is provided by a GASONeC method [21], in which the node residual energy, the expected energy expenditure, the distance to the BS, and the number of sensor nodes in the vicinity are considered. Balancing these factors is the key to dividing sensor nodes into clusters and designating surrogate sensor nodes as CHs, which contributes to the maximizing of WSN longevity. However, its computational complexity increases dramatically for a large size of iteration generations. In this paper, the DHCO algorithm aims to balance the energy consumption of sensor nodes and improve WSN longevity by synthetically taking into account the node energy and the node distance properties. Specially, the DHCO algorithm utilizes the node distance property to construct a hierarchical network structure that is the vital foundation of the feasible routing set for each sensor node, with the feasible routing set supplying a solution space to obtain the optimal route in consideration of the node energy expenditure.

## 3. The System Model

### 3.1. Network Model

In the grid-based development field, there are great many randomly distributed sensor nodes. The network topology including all sensor nodes and the BS builds a graph $G\left(V,E\right)$, where $V=\left\{v_{0},v_{1},\cdots ,v_{i},\cdots ,v_{n}\right\}$ is the node set, $v_{0}$ is the BS, and $E=\left\{e_{1},\cdots ,e_{j},\cdots ,e_{m}\right\}$ is the communication link set of sensor nodes. Then, we make some reasonable and realistic assumptions regarding the WSNs [21,22]:

(1) The BS and all sensor nodes are stationary after deployment, and are equipped with a Global Positioning System (GPS) unit. Hence, these nodes are location-aware.
(2) All properties for each sensor node are identical, while the BS is manually maintained and has enough energy to support continuous operations, with its energy denoted as $\varepsilon_{0}$.
(3) Provided with sufficient energy, each node can control the transmission power according to the distance between the transmitter and the receiver.
(4) The amount of transmission data is exactly equal for each sensor node of valid routes.

At the transmission round *t*, the residual energy of all sensor nodes is denoted as $\Psi \left(t\right)={\left[\varepsilon_{0},\varepsilon_{1}\left(t\right),\cdots ,\varepsilon_{i}\left(t\right),\cdots ,\varepsilon_{n}\left(t\right)\right]}^{T}$, while their corresponding initial node energy is denoted as $\Psi \left(0\right)={\left[\varepsilon_{0},\varepsilon_{1}\left(0\right),\cdots ,\varepsilon_{i}\left(0\right),\cdots ,\varepsilon_{n}\left(0\right)\right]}^{T}$, where *t* is the time scale in terms of transmission rounds of the WSNs [21]. The neighboring communication link between two sensor nodes is determined by the communication radius $r_{s}$ that is defined as follows [22]:

(1) $$r_{s}=\left[1-\frac{d_{\max}-d\left(v_{i},v_{0}\right)}{\mu \left(d_{\max}-d_{\min}\right)}\right]r_{\max}\;,\;i\in \left\{1,2,\cdots ,n\right\}\;$$

where $r_{max}$ is the maximum communication radius and is defined as the width of the sensing field, and $d_{max}$ and $d_{min}$ represent the maximum and minimum distances between the sensor node and the BS, respectively. The distance $d\left(v_{i},v_{0}\right)$ denotes the distance between $v_{i}$ and the BS $v_{0}$, and the variable μ can be adjusted according to the real environment. Extensive experiments show that a smaller value of μ easily damages the network connectivity, and that a larger value of μ is prone to cause packet collisions among sensor nodes. According to the mathematical analysis of Equation (1), for the variable μ there exists a point $\mu^{*}$ at which the increasing trend of $r_{s}$ changes dramatically. The communication radius $r_{s}$ increases rapidly before the point $\mu^{*}$, while it increases slowly after the point $\mu^{*}$. In order to minimize packet collisions among the sensor nodes and to guarantee the transmission quality of WSNs as much as possible [22], we conduct diverse experiments for different values of μ within the numerical region $\left[\mu^{*}-\Delta \mu ,\;\mu^{*}+\Delta \mu \right]$ to obtain the optimal communication radius $r_{s}^{*}$, where Δμ=0.5 is an offset boundary of the trim parameter that can be adjusted.

For the sensor node $v_{i}$, its neighboring nodes’ set comprises sensor nodes within the optimal communication radius $r_{s}^{*}$. Meanwhile, the node residual energy must accommodate the basic requirement of effective communication; otherwise, the sensor node is defined as a "dead node" and is excluded from its corresponding neighboring nodes’ sets. The neighboring communication link between $v_{i}$ and $v_{j}$ with the distance $d(v_{i},v_{j})$ is denoted by

(2) $$m_{ij}=\left\{\begin{array}{c} 1;\;d\left(v_{i},v_{j}\right)\leq r_{s}^{*},\;\varepsilon_{i}\left(t\right)>\hat{\varepsilon },\;\varepsilon_{j}\left(t\right)>\hat{\varepsilon }\\ 0;\;otherwise \end{array}\right.$$

where i,j∈{0,1,2,⋯,n}, and $\hat{\varepsilon }$ is the minimum residual node energy that pledges communication. All neighboring communication links $m_{ij}$ construct an adjacency matrix:

(3) $$M\left(G\right)=\left[\begin{array}{ccccc} m_{00} & m_{01} & m_{02} & \cdots & m_{0n}\\ m_{10} & m_{11} & m_{12} & \cdots & m_{1n}\\ \vdots & \vdots & \vdots & \vdots & \vdots \\ m_{n0} & m_{n1} & m_{n2} & \cdots & m_{nn} \end{array}\right]$$

where we define $m_{ii}=0$. Moreover, each sensor node may belong to one or more neighboring nodes’ sets, and can calculate we $M\left(G\right)$ independently.

### 3.2. Sensor Energy Model

**Figure 1:**
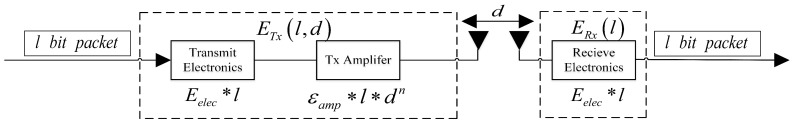
Radio energy dissipation model.

The radio model follows that in [23], as shown in Figure 1. Assuming that the wireless channel is completely symmetrical, the energy consumption of transmitting a message over the back and forth route between $v_{i}$ and $v_{j}$ is equal. If the communication distance $d(v_{i},v_{j})$ from $v_{i}$ to $v_{j}$ is larger than the threshold distance $d_{0}$, i.e., $d\left(v_{i},v_{j}\right)\geq d_{0}$, the multipath fading ($d^{4}\left(v_{i},v_{j}\right)$ consumption loss) channel model is used; otherwise, the free space ($d^{2}\left(v_{i},v_{j}\right)$ consumption loss) channel model is chosen. Then, for transmitting an *l*-bits message over $d(v_{i},v_{j})$, the energy consumption is given by

(4) $$\begin{array}{c} E_{T}\left(l,d(v_{i},v_{j})\right)=\left\{\begin{array}{c} l*E_{elec}+l*\varepsilon_{fs}*d^{2}\left(v_{i},v_{j}\right),\;\;\;d(v_{i},v_{j})<d_{0}\\ l*E_{elec}+l*\varepsilon_{mp}*d^{4}\left(v_{i},v_{j}\right),\;\;d\left(v_{i},v_{j}\right)\geq d_{0} \end{array}\right. \end{array}$$

and the energy consumption for receiving the *l*-bits message over $d(v_{i},v_{j})$ is given by

(5) $$E_{R}\left(l\right)=l*E_{elec}$$

Above, $E_{elec}$ denotes the transmitting circuit loss, which depends on the digital coding, modulation, filtering, and spreading of the signal. The energy coefficients for power amplification in two different channel models are denoted by $\varepsilon_{fs}*d^{2}\left(v_{i},v_{j}\right)$ and $\varepsilon_{mp}*d^{4}\left(v_{i},v_{j}\right)$, respectively.

## 4. Proposed Protocol

In this Section, the DHCO algorithm is described in detail. Differently to conventional WSN routing protocols (i.e., LEACH, HEED, etc.) for selecting the CH or the next hop based on the node energy or the node distance properties, or a combination of these two properties, the DHCO algorithm constructs a hierarchical network structure according to the node distance property, and then utilizes the combinatorial optimization theory to establish the feasible routing set for each sensor node. The feasible routing set for each sensor node, moreover, is equivalent to a solution space. Next, taking advantage of the maximum–minimum criterion, the optimal route is obtained from the solution space on the basis of the node energy property, which ultimately evenly distributes network traffic load to balance the energy consumption of sensor nodes and improve WSN longevity. Therefore, the process of obtaining the optimal route can be formulated as a combinatorial optimization problem.

### 4.1. Constructing the Hierarchical Network Structure

In order to construct a hierarchical network structure, we assume that each sensor node is layered and belongs to a specific node set according to the adjacency matrix $M\left(G\right)$. Specifically, the BS $v_{0}$ is the unique element of the first-layer subset $V_{1}$; the second-layer subset $V_{2}$ comprises the neighboring sensor nodes of the BS $v_{0}$, and the neighboring sensor nodes that are adjacent to $V_{2}$ constitute the third-layer subset $V_{3}$. This process repeats itself until all sensor nodes are stratified. Finally, a hierarchical network structure $T\left(V_{T},E_{T}\right)$ for all nodes is built, where $V_{T}=V$, $E_{T}$ is the edge set with the edge representing the neighboring communication link between sensor nodes of adjacent layers, and $E_{T}\subset E$. The hierarchical network structure $T\left(V_{T},E_{T}\right)$ is denoted as

(6) $$\left\{\begin{array}{c} V_{1}=\left\{v_{0}\right\}\\ V_{2}=\left\{v_{p}\in V|m_{p0}=1,\;e_{p0}\in E_{T}\right\}\\ \cdots \cdots \\ V_{k}=\left\{v_{p}\in V|v_{q}\in V_{k-1},m_{pq}=1,\;e_{pq}\in E_{T}\right\}\\ \cdots \cdots \\ V_{K}=\left\{v_{p}\in V|v_{q}\in V_{K-1},m_{pq}=1,\;e_{pq}\in E_{T}\right\} \end{array}\right.$$

where $V_{k}$ is the *k*-layer sensor node subset of $T\left(V_{T},E_{T}\right)$. Meanwhile, $V_{T}=V_{1}\cup \cdots \cup V_{k}\cup \cdots \cup V_{K}$, $V_{s}\cap V_{t}=\emptyset$ for any s≠t and $s,t\in \left\{1,\cdots ,k,\cdots ,K\right\}$.

### 4.2. Establishing the Feasible Routing Set

The hierarchical network structure $T\left(V_{T},E_{T}\right)$ is the basis of establishing the feasible routing set for each sensor node. Each feasible routing set is composed of a great number of valid routes that can be constructed by adding sensor nodes, which could participate in routes layer by layer until the BS $v_{0}$ is added. Next, we give an example that displays this process from the transmitter $v_{i}$ to the BS $v_{0}$. Assume that $v_{i}\in V_{k(i)}$ and k(i) is the hierarchical number of $v_{i}$ in $T(V_{T},E_{T})$ and is determined by the location of $v_{i}$ regarding $v_{0}$. In order to establish a feasible routing set $R_{i}$ for the transmitter $v_{i}$, we first build a hierarchical network structure $T_{i}\left(V_{T}^{\left(i\right)},E_{T}^{\left(i\right)}\right)$ on the basis of $T(V_{T},E_{T})$. The hierarchical network structure $T_{i}\left(V_{T}^{\left(i\right)},E_{T}^{\left(i\right)}\right)$ for the transmitter $v_{i}$ is similar to $T(V_{T},E_{T})$ and is denoted as

(7) $$\left\{\;\begin{array}{c} V_{1}^{\left(i\right)}=\left\{v_{0}\right\}\\ \cdots \cdots \\ V_{j}^{\left(i\right)}\;=\;\left\{v_{p}\in V_{T}^{\left(i\right)}|v_{q}\in V_{j-1}^{\left(i\right)},m_{pq}=1,\;e_{pq}\in E_{T}^{\left(i\right)}\right\}\;\;\\ \cdots \cdots \\ V_{k\left(i\right)}^{\left(i\right)}=\left\{v_{i}\right\} \end{array}\right.\;\;,\;\;$$

where $V_{j}^{\left(i\right)}\;\subset \;V_{j}$, $V_{T}^{\left(i\right)}\;=\;V_{1}^{\left(i\right)}\cup \cdots \cup V_{j}^{\left(i\right)}\cup \cdots \cup V_{k\left(i\right)}^{\left(i\right)}$, and $E_{T}^{\left(i\right)}\subset E_{T}$.

The hierarchical network structure $T_{i}\left(V_{T}^{\left(i\right)}E_{T}^{\left(i\right)}\right)$ is the optimal network structure for $v_{i}$, as well as the foundation for establishing the feasible routing set $R_{i}$. Each time we select one sensor node from the sensor node subset $V_{j}^{\left(i\right)}$, we also perform the same operation for all other layers of $V_{T}^{\left(i\right)}$. These selected sensor nodes will construct a valid route from the transmitter $v_{i}$ to the BS $v_{0}$, i.e., the *p*th valid route of the feasible routing set $R_{i}$, denoted by

(8) $$r_{p}^{\left(i\right)}=\left[v_{\left(p,k(i)\right)}^{\left(i\right)}\to \cdots \to v_{\left(p,j\right)}^{\left(i\right)}\to \cdots \to v_{\left(p,2\right)}^{\left(i\right)}\to v_{\left(p,1\right)}^{\left(i\right)}\right]$$

where $v_{\left(p,j\right)}^{\left(i\right)}\in V_{j}^{\left(i\right)}$.

To unify formation, let $v_{\left(p,k(i)\right)}^{\left(i\right)}$ represent $v_{i}$ and $v_{\left(p,1\right)}^{\left(i\right)}$ represent $v_{0}$. This process repeats, and all valid routes such as $r_{p}^{\left(i\right)}$ establish the feasible routing set $R_{i}$ that is shown as

(9) $$\begin{array}{c} R_{i}=\left\{r_{p}^{\left(i\right)}|1\leq p\leq N_{i},\;p\in N^{+}\right\}=\left\{\begin{array}{c} \left[\begin{array}{ccccccc} \;\;v_{\left(1,k\left(i\right)\right)}^{\left(i\right)} & \;\;\to & \;\;\cdots & v_{\left(1,j\right)}^{\left(i\right)} & \to & \;\;\cdots & v_{\left(1,1\right)}^{\left(i\right)}\;\; \end{array}\right]\\ \vdots \\ \left[\begin{array}{ccccccc} \;\;v_{\left(p,k\left(i\right)\right)}^{\left(i\right)} & \;\;\to & \;\;\cdots & v_{\left(p,j\right)}^{\left(i\right)} & \to & \;\;\cdots & v_{\left(p,1\right)}^{\left(i\right)}\;\; \end{array}\right]\\ \vdots \\ \;\;\left[\;\;\begin{array}{ccccccc} v_{\left(N_{i},k\left(i\right)\right)}^{\left(i\right)} & \;\;\to \;\; & \;\;\cdots \;\; & v_{\left(N_{i},j\right)}^{\left(i\right)} & \;\;\to \;\; & \cdots \;\; & v_{\left(N_{i},1\right)}^{\left(i\right)} \end{array}\;\;\right] \end{array}\;\;\;\right\} \end{array}\;$$

where $N_{i}=\left|V_{1}^{\left(i\right)}\right|\times \cdots \times \left|V_{j}^{\left(i\right)}\right|\times \cdots \times \left|V_{k(i)}^{\left(i\right)}\right|$ denotes the number of valid routes in $R_{i}$.

### 4.3. Obtaining the Optimal Route

The feasible routing set $R_{i}$ including many valid routes provides a solution space in which we obtain the optimal route from the transmitter $v_{i}$ to the BS $v_{0}$ according to the node energy property. Therefore, the procedure of obtaining the optimal route can be formulated as a combinatorial optimization problem. As we know, the bucket effect refers to the law that the shortest piece of wood constrains the amount of water in a bucket; the same principle also applies to obtain the optimal route. Hence, the sensor node with the minimal residual energy along a valid route limits the transmission capacity of the route. In order to achieve energy usage evenly, the maximum–minimum criterion is used to obtain the especial sensor node defined by $v_{\left(p^{*},j^{*}\right)}^{\left(i\right)}$, which has the minimum residual energy for the sensor nodes along the optimal route $r_{p^{*}}^{\left(i\right)}$, and whose routing transmission capacity is maximized in the feasible routing set $R_{i}$. At the transmission round *t*, the residual energy of the sensor nodes in the feasible routing set $R_{i}$ can build an energy matrix named $\Psi_{i}\left(t\right)$ that is shown as

(10) $$\begin{array}{c} \;\;\;\;\Psi_{i}\left(t\right)\;=\;\;\left[\;\;\;\begin{array}{ccccc} \varepsilon_{\left(1,k(i)\right)}^{\left(i\right)}\left(t\right) & \;\cdots & \varepsilon_{\left(1,j\right)}^{\left(i\right)}\left(t\right) & \cdots & \varepsilon_{\left(1,1\right)}^{\left(i\right)}\left(t\right)\\ \vdots & \vdots & \vdots & \vdots & \vdots \\ \varepsilon_{\left(p,k(i)\right)}^{\left(i\right)}\left(t\right) & \cdots & \varepsilon_{\left(p,j\right)}^{\left(i\right)}\left(t\right) & \cdots & \varepsilon_{\left(p,1\right)}^{\left(i\right)}\left(t\right)\\ \vdots & \vdots & \vdots & \vdots & \vdots \\ \varepsilon_{\left(N_{i},k\left(i\right)\right)}^{\left(i\right)}\left(t\right) & \;\cdots \;\; & \;\varepsilon_{\left(N_{i},j\right)}^{\left(i\right)}\left(t\right) & \;\cdots \;\; & \varepsilon_{\left(N_{i},1\right)}^{\left(i\right)}\left(t\right) \end{array}\;\;\;\right]\;\; \end{array}\;\;\;\;$$

where $\varepsilon_{\left(p,j\right)}^{\left(i\right)}\left(t\right)$ represents the residual energy of the sensor node $v_{\left(p,j\right)}^{\left(i\right)}$ at the transmission round *t*.

In order to obtain the especial sensor node $v_{\left(p^{*},j^{*}\right)}^{\left(i\right)}$, we calculate the transmission capacity for each route based on the node residual energy, and then obtain the route with the maximal transmission capacity from the feasible routing set $R_{i}$. The procedures of solving for the especial sensor node $v_{\left(p^{*},j^{*}\right)}^{\left(i\right)}$ and the optimal route $r_{p^{*}}^{\left(i\right)}$ for the transmitter $v_{i}$ are modeled as follows:

(11) $$\begin{array}{c} \left(p^{*},j^{*}\right)=\arg\;\max_{p}\left\{\min_{j}\;\varepsilon_{\left(p,j\right)}^{\left(i\right)}\left(t\right)\right\}=\arg\;\max_{p}\;\varepsilon_{\left(p,j^{*}\right)}^{\left(i\right)}\left(t\right);\\ p\in \left\{1,2,\cdots ,N_{i}\right\},\;j\in \left\{1,2,\cdots ,k\left(i\right)\right\} \end{array}$$

The value of the subscript $p^{*}$ can locate the optimal route $r_{p^{*}}^{\left(i\right)}$, while values of the subscripts $p^{*}$ and $j^{*}$ can jointly locate the especial sensor node $v_{\left(p^{*},j^{*}\right)}^{\left(i\right)}$. Thus the optimal route for the transmitter $v_{i}$ is given by

(12) $$r_{p^{*}}^{\left(i\right)}=\left[v_{\left(p^{*},k\left(i\right)\right)}^{\left(i\right)}\to \cdots \to v_{\left(p^{*},j^{*}\right)}^{\left(i\right)}\to \cdots \to v_{\left(p^{*},2\right)}^{\left(i\right)}\to v_{\left(p^{*},1\right)}^{\left(i\right)}\right]$$

Finally, at the transmission round *t*, the transmitter $v_{i}$ sends information gathered to the BS $v_{0}$ in line with $r_{p^{*}}^{\left(i\right)}$, and the sensor nodes along the optimal route $r_{p^{*}}^{\left(i\right)}$ will decrease in energy based on Equations (4) and (5). Then the remaining energy of the sensor nodes along the optimal route $r_{p^{*}}^{\left(i\right)}$ is given by

(13) $$f\left(\Psi \left(t\right),r_{p^{*}}^{\left(i\right)}\right)\;\;=\;\;\;\;\;\begin{array}{c} \left[\;\;\begin{array}{c} \varepsilon_{\left(p^{*},k\left(i\right)\right)}^{\left(i\right)}\left(t\right)-0-E_{T}\left(l,d\left(v_{\left(p^{*},k\left(i\right)\right)}^{\left(i\right)},v_{\left(p^{*},k\left(i\right)-1\right)}^{\left(i\right)}\right)\right)\\ \vdots \\ \varepsilon_{\left(p^{*},j^{*}\right)}^{\left(i\right)}\left(t\right)-E_{R}\left(l\right)-E_{T}\left(l,d\left(v_{\left(p^{*},j^{*}\right)}^{\left(i\right)},v_{\left(p^{*},j^{*}-1\right)}^{\left(i\right)}\right)\right)\\ \vdots \\ \varepsilon_{\left(p^{*},2\right)}^{\left(i\right)}\left(t\right)-E_{R}\left(l\right)-E_{T}\left(l,d\left(v_{\left(p^{*},2\right)}^{\left(i\right)},v_{\left(p^{*},1\right)}^{\left(i\right)}\right)\right)\\ \varepsilon_{\left(p^{*},1\right)}^{\left(i\right)}\left(t\right)-E_{R}\left(l\right)-0 \end{array}\;\;\right] \end{array}\;\;\;$$

Through procedures described in Section 4.1, Section 4.2 and Section 4.3, the transmitter $v_{i}$ successfully sends information gathered to the BS $v_{0}$, and other sensor nodes also do the same. After all sensor nodes send information gathered to the BS $v_{0}$ once, the process repeats, and the next transmission round begins again [21,24].

## 5. Performance Evaluation

In this Section, we provide various simulation experiments to demonstrate the effectiveness and superiority of the DHCO algorithm, in comparison—from multiple perspectives—with state-of-the-art WSN routing algorithms such as LEACH [11], HEED [10], GASONeC [21], and DCFR [15], that consist of the number of sensor nodes and the width of the sensing field. For the sake of acquiring a higher level of confidence, we conducted 600 experiments per each experimental scenario with randomly distributed sensor nodes, and calculated their statistical averages as the results. Evaluating criterions for the WSN longevity were the transmission rounds and percentages of live sensor nodes that had different definitions. The performance aspect of the percentage of live sensor nodes was the case of how many sensor nodes were alive at the transmission round *t*; the more sensor nodes that were alive, the better the algorithm performed. Another performance aspect was given by the statistics for the transmission rounds of diverse experimental scenarios in WSNs; the more transmission rounds there were, the better the algorithm performed. More details are presented as follows.

### 5.1. Experimental Setup

**Figure 2:**
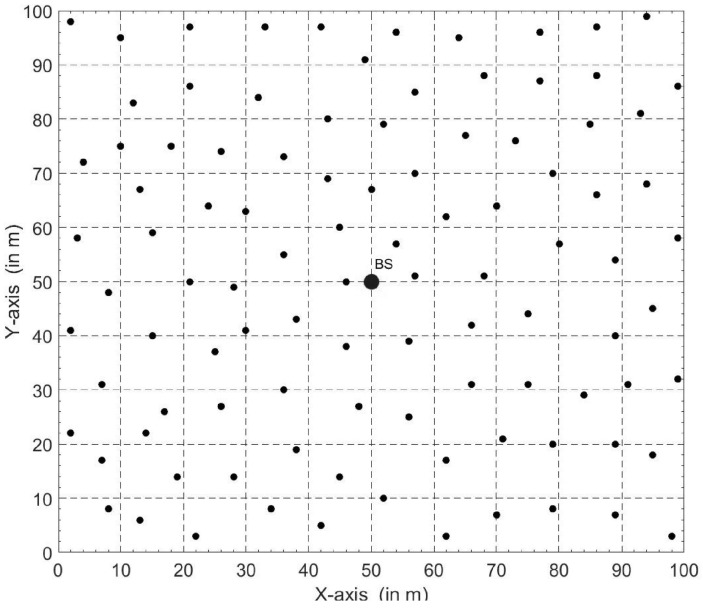
One-hundred sensor nodes’ random distribution in the deployment scenario of a 100 m × 100 m square region, and the coordinate (50, 50) of the BS.

For the simulation experiments, algorithms including DHCO, LEACH, HEED, GASONeC, and DCFR ran on diverse deployment scenarios, for which sensor nodes were randomly distributed and the BS was located at a certain position. For example, in Figure 2, the simulation experiments had a deployment scenario of 100 sensor nodes randomly distributed in a 100m×100m square, with the BS located at the center of the sensing field (coordinate (50, 50)). Moreover, diverse simulation experiments of DHCO, LEACH, HEED, GASONeC, and DCFR algorithms were implemented in MATLAB 2015b, and were conducted on a computer with an Intel Core i7-4790 CPU (Lenovo, Beijing, China) of 3.60 GHz and 8G memory, and a Windows 7 operating system.

In general, the radio wave propagation was highly variable and difficult to be modeled. Thus, as for the radio model used in [23], our radio model was also a simplified model, in which the parameters that were used for computations in the simulation experiments refer to [21,22,25] and are listed in Table 1.

### 5.2. Node Energy Consumption and Wireless Sensor Network Longevity for the Dynamic Hierarchical Protocol Based on Combinatorial Optimization Algorithm

**Figure 3:**
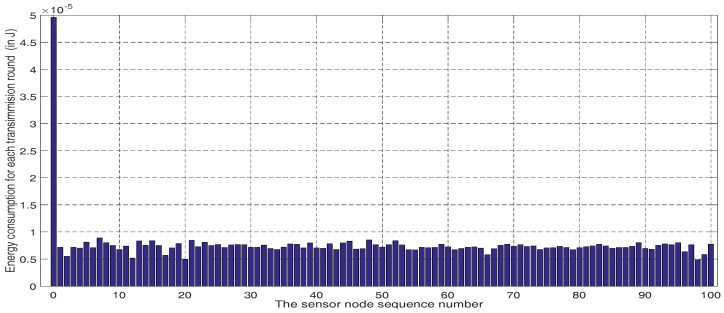
Averages of sensor nodes’ energy consumption for one transmission round in the dynamic hierarchical protocol based on combinatorial optimization (DHCO) algorithm.

**Figure 4:**
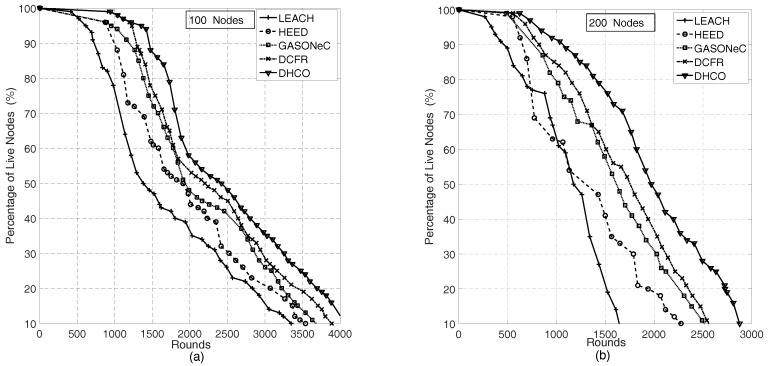
Percentage of live sensor nodes with sensor nodes of a random distribution for various algorithms in the deployment scenario of a 100m×100m square region and the coordinate (50, 50) of the BS: (**a**) 100 sensor nodes of a random distribution, and (**b**) 200 sensor nodes of a random distribution.

In this subsection, simulation experiments were conducted to compare the percentage of live sensor nodes of the DHCO algorithm with that of LEACH, HEED, GASONeC, and DCFR algorithms for different transmission rounds in WSNs. The sensing field was set to a 100m×100m square region and the BS was located at coordinate (50, 50); this was used to measure two different deployment scenarios separately: 100 sensor nodes with a random distribution and 200 sensor nodes with a random distribution. For each transmission round, every sensor node acted as a transmitter or a router, and consumed energy. In the DHCO algorithm, the statistical average of the nodes’ energy consumption for one transmission round was calculated, and is depicted in Figure 3; the energy consumption of the BS $v_{0}$ was extremely large, which resulted from the fact that the BS is responsible for receiving information about all sensor nodes, and each sensor node is only responsible for sending or receiving information about itself or several of its nearby neighboring sensor nodes. We found that the energy consumptions for all sensor nodes were close, owning to the balanced energy consumption via the DHCO algorithm. As the transmission round of the WSNs went on, the simulation experiments presented illustrative numerical results to assess the performances of the WSN routing algorithms, and these are shown in Figure 4.

Figure 4 indicates that, for the same transmission rounds, the curve of the DHCO algorithm corresponding to the percentage of live sensor nodes evidently always overtops that of the LEACH, HEED, GASONeC, and DCFR algorithms, and the number of sensor nodes in the WSNs has an effect on the transmission rounds. Hence, the DHCO algorithm shows better performance compared to the LEACH, HEED, GASONeC, and DCFR algorithms. This can be explained by the fact that the route with the maximal transmission capacity, being limited by the sensor node with the minimal residual energy rather than the better sensor node (i.e., the node with a greater node residual energy or shorter distance to its neighbors), is the selected object. Thus, the optimal route, holding sensor nodes with greater residual energy, has more chance to be chosen, and sensor nodes with low residual energy are protected, which can evenly distribute the network traffic load to all sensor nodes and improve the WSN longevity.

### 5.3. Wireless Sensor Network Longevity versus the Width of the Sensing Field and the Number of Sensor  Nodes

**Figure 5:**
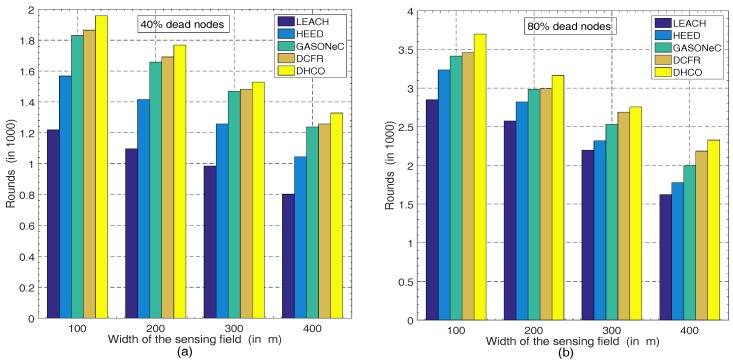
Comparison of transmission rounds with different dead sensor nodes for various algorithms in the deployment scenarios of 100 sensor nodes of a random distribution and the coordinate (50, 50) of the BS: (**a**) at the 40% dead-sensor-nodes level for different widths of the sensing field, and (**b**) at the 80% dead-sensor-nodes level for different widths of the sensing field.

**Figure 6:**
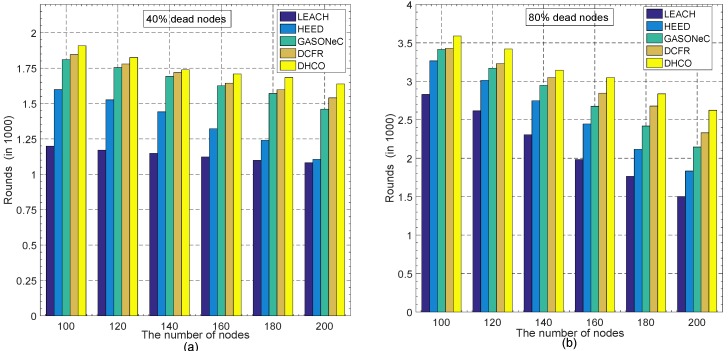
Comparison of transmission rounds with different dead sensor nodes for various algorithms in the deployment scenarios of the 100m×100m square region and the coordinate (50, 50) of the BS: (**a**) at the 40% dead-sensor-nodes level for different numbers of sensor nodes, and (**b**) at the 80% dead-sensor-nodes level for different numbers of sensor nodes.

The number of sensor nodes and the width of the sensing field are critical factors influencing the energy consumption of sensor nodes and the WSN longevity. For diverse widths of the sensing fields and various numbers of sensor nodes, we carried out several simulation experiments, whose results are exhibited vividly by Figure 5 and Figure 6, respectively.

In the deployment scenario of 100 randomly distributed sensor nodes and the BS located at the coordinate (50, 50), transmission rounds decreased with the increasing width of the sensing field, according to Figure 5a, showing 40% dead sensor nodes, and Figure 5b, showing 80% dead sensor nodes, which resulted from the increase of the transmission distance between the sensor nodes. Simultaneously, according to Figure 6a, showing 40% dead sensor nodes, and Figure 6b, showing 80% dead sensor nodes, transmission rounds also decreased with the increasing number of sensor nodes in the deployment scenario, where the region was a 100m×100m square and the BS was located at the coordinate (50, 50). Although the effect of the reduction resulting from the increasing number of sensor nodes was not significant, the decreasing trend still existed. In WSNs, because the increase in the number of sensor nodes brings about a much greater amount of information gathered, this will produce a decline in transmission rounds. The experimental results are apparent and can be supported perfectly by Equation (4). Thus, the figures (including Figure 5 and Figure 6) indicate that, regardless of various numbers of sensor nodes or diverse widths of the sensing field in WSNs, the DHCO algorithm always performs more transmission rounds in comparison with state-of-the-art WSN routing algorithms such as LEACH, HEED, GASONeC, and DCFR. Therefore, the DHCO algorithm has a better effectiveness and scalability than these WSN routing algorithms.

### 5.4. Efficiency and Computational Complexity Discussion

In the DHCO algorithm, the most time-consuming procedures were to construct the hierarchical network structure for all sensor nodes and to establish the feasible routing set for each sensor node. For each transmission round, the mean time (in s) and the standard deviation of the computational complexity for the DHCO algorithm were accurately calculated. Table 2 elaborates the statistical results of the computational complexity for the diverse deployment scenarios. We found that the mean time and the standard deviation of the computational complexity were close for all deployment scenarios, except that the mean time rose as the number of sensor nodes increased.

**Figure 7:**
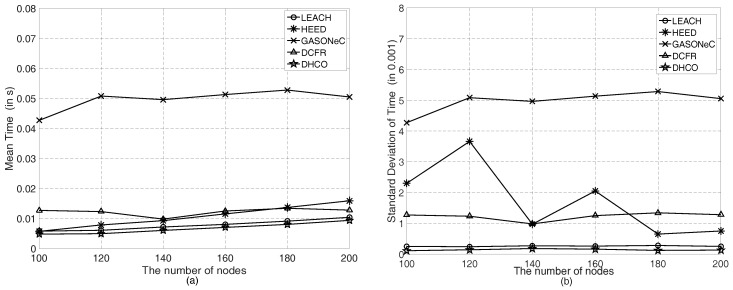
Comparison of the mean time and standard deviation of the computational complexity for each transmission round in the deployment scenarios of the 100m×100m square region and the coordinate (50, 50) of the BS: (**a**) mean time for diverse numbers of sensor nodes, and (**b**) standard deviation of the computational complexity for diverse numbers of sensor nodes.

In consideration of various numbers of sensor nodes, Figure 7 exhibits the comparison of the mean time and standard deviation of the computational complexity for one transmission round in the deployment scenario, in which the region was a 100m×100m square and the BS was located at the coordinate (50, 50). In detail, Figure 7a shows that the mean time of the DHCO algorithm for various numbers of sensor nodes was lower than that of state-of-the-art WSN routing algorithms such as LEACH, HEED, GASONeC, and DCFR, and Figure 7b shows that the standard deviation of the computational complexity for the DHCO algorithm was also lower than that of the state-of-the-art WSN routing algorithms. Owing to the fact that the GASONeC algorithm employs more chromosomes in any generation and that more offsprings are created, its computational complexity was the highest. Moreover, according to the hierarchical network structure $T\left(V_{T},E_{T}\right)$, the DHCO algorithm only needs to choose the optimal route from its established feasible routing set, which contributed to decreasing the scale of the routing optimization problem and the mean computational time. Differently to many conventional WSN routing algorithms (i.e., LEACH, HEED, etc.) that form clusters by using random factors, the DHCO algorithm significantly reduces the effect of randomness, so that the standard deviation of the computational complexity of the DHCO algorithm is the lowest. We found that the DHCO algorithm had a lower computational complexity than the state-of-the-art WSN routing algorithms LEACH, HEED, GASONeC, and DCFR.

In Section 5.2, Section 5.3 and Section 5.4, many different strategies were adopted to compare the performance of the DHCO algorithm with that of the LEACH, HEED, GASONeC, and DCFR algorithms. All experimental results indicated that the DHCO algorithm has superior performance over state-of-the-art WSN routing algorithms. These state-of-the-art WSN routing algorithms employ the clustering mechanism to improve WSN longevity, but fail to solve the issue of unevenly distributed CHs well, which has a poor impact on their performance. Differently to state-of-the-art WSN routing algorithms, the DHCO algorithm utilizes all sensor nodes and the BS to construct the hierarchical network structure, and employs the combinatorial optimization theory to establish the feasible routing set for each sensor node, taking advantage of the maximum–minimum criterion to obtain their optimal routes based on the node residual energy; it significantly reduces the effect of randomness and ensures each sensor node has an equal opportunity to participate in the network routing operation, which contributes to a balanced energy consumption of the sensor nodes, improves WSN longevity, and reduces the computational complexity of the algorithm.

## 6. Conclusions

In this paper, we propose a DHCO algorithm to balance the energy consumption of sensor nodes and to improve WSN longevity. Compared with state-of-the-art WSN routing algorithms, including LEACH, HEED, GASONeC, and DCFR, the DHCO algorithm constructs a hierarchical network structure based on the node distance property and establishes the feasible routing set that provides the solution space; these are the foundations for the formation of a combinatorial optimization problem. Taking into account the node energy property, the DHCO algorithm takes advantage of the maximum minimum criterion to obtain the optimal route for each sensor node from their corresponding solution spaces. Diverse experimental results demonstrated that the DHCO algorithm is feasible and has superior performance over the existing WSN routing algorithms with respect to balancing the energy consumption of sensor nodes and improving WSN longevity. Finally, as future work, this can be extended for the WSNs with sensor nodes that follow a mobility pattern; this is a significant subject differing from the current WSNs, for which the sensor nodes follow a mobility pattern.

## Figures and Tables

**Table 1:** The algorithms’ setting parameters.

Properties	Values
Initial node energy	0.5 J
Electronics energy, $E_{elec}$	50 nJ/bit
Consumption loss for $d^{2}$, $\varepsilon_{fs}$	10 pJ/bit/m${}^{2}$
Consumption loss for $d^{4}$, $\varepsilon_{amp}$	0.0013 pJ/bit/m${}^{4}$
Data aggregation energy	50 nJ/bit/signal
Packet size, *l*	400 bit
The optimal communication radius, $r_{s}$	40 m
The threshold distance, $d_{0}$	75 m
The minimum residual node energy, $\hat{\varepsilon }$	5μJ
The initial probability *p* of being a CH	0.05
The maximum number of iterations in HEED	12
The population size in GASONeC	30
The generation size in GASONeC	30
The crossover probability in GASONeC	0.8
The mutation probability in GASONeC	0.006
Duty cycle γ in DCFR	10%
Duration of a data period $T_{r}$ in DCFR	10 s
Energy consumption rate for idle listening $E_{idle}$ in DCFR	0.88 mJ/s

**Table 2:** Statistical Time of Computational Complexity for Various Deployment Scenarios.

Width of Square Region	Location of BS	Number of Nodes	Mean Time	Standard Deviation
100 m	(50 m, 50 m)	100	0.00479 s	0.00011
100 m	(50 m, 50 m)	120	0.00494 s	0.00014
100 m	(50 m, 50 m)	140	0.00603 s	0.00018
100 m	(50 m, 50 m)	160	0.00703 s	0.00016
100 m	(50 m, 50 m)	180	0.00803 s	0.00012
100 m	(50 m, 50 m)	200	0.00937 s	0.00013
100 m	(100 m, 100 m)	100	0.00417 s	0.00015
100 m	(150 m, 150 m)	100	0.00407 s	0.00014
100 m	(200 m, 200 m)	100	0.00393 s	0.00009
100 m	(250 m, 250 m)	100	0.00408 s	0.00012
200 m	(50 m, 50 m)	100	0.00409 s	0.00015
300 m	(50 m, 50 m)	100	0.00389 s	0.00008
400 m	(50 m, 50m)	100	0.00411 s	0.00011
